# Antimicrobial Resistance and Spread of Multi Drug Resistant *Escherichia coli* Isolates Collected from Nine Urology Services in the Euregion Meuse-Rhine

**DOI:** 10.1371/journal.pone.0047707

**Published:** 2012-10-17

**Authors:** Christina F. M. van der Donk, Jeroen H. B. van de Bovenkamp, Els I. G. B. De Brauwer, Patrick De Mol, Karl-Heinz Feldhoff, Wiltrud M. Kalka-Moll, Sita Nys, Inge Thoelen, Thera A. M. Trienekens, Ellen E. Stobberingh

**Affiliations:** 1 Maastricht University Medical Centre, Department of Medical Microbiology, Maastricht, The Netherlands; 2 Maastricht University, CAPHRI School for Public Health and Primary Care, Maastricht, The Netherlands; 3 PAMM, Laboratory of Medical Microbiology, Veldhoven, The Netherlands; 4 Atrium Medical Centre, Department of Medical Microbiology, Heerlen, The Netherlands; 5 Centre Hospitalier Universitaire de Liège, Laboratoire de Microbiologie, Liège, Belgium; 6 Gesundheitsamt des Kreises Heinsberg, Heinsberg, Germany; 7 Medical Care Centre Dr. Stein and Colleagues, Moenchengladbach, Germany; 8 Jessa Hospital, Campus Virga Jesse, Department of Clinical Biology, Hasselt, Belgium; 9 General Hospital Vesalius, Department of Clinical Biology, Tongeren, Belgium; 10 VieCuri Medical Centre, Department of Medical Microbiology, Venlo, The Netherlands; Charité, Campus Benjamin Franklin, Germany

## Abstract

We determined the prevalence and spread of antibiotic resistance and the characteristics of ESBL producing and/or multi drug resistant (MDR) *Escherichia coli* isolates collected from urine samples from urology services in the Euregio Meuse-Rhine, the border region of the Netherlands (n = 176), Belgium (n = 126) and Germay (n = 119). Significant differences in resistance between the three regions were observed. Amoxicillin-clavulanic acid resistance ranged from 24% in the Netherlands to 39% in Belgium (p = 0.018), from 20% to 40% (p<0.004) for the fluoroquinolones and from 20% to 40% (p = 0.018) for the folate antagonists. Resistance to nitrofurantoin was less than 5%. The prevalence of ESBL producing isolates varied from 2% among the Dutch isolates to 8% among the German ones (p = 0.012) and were mainly CTX-M 15. The prevalence of MDR isolates among the Dutch, German and Belgian isolates was 11%, 17% and 27%, respectively (p< = 0.001 for the Belgian compared with the Dutch isolates). The majority of the MDR and ESBL producing isolates belonged to ST131. This study indicates that most antibiotics used as first choice oral empiric treatment for UTIs (amoxicillin-clavulanic acid, fluoroquinolones and folate antagonists) are not appropriate for this purpose and that MDR strains such as CTX-M producing ST131 have spread in the entire Euregion. Our data stress the importance of ward specific surveillance to optimize empiric treatment. Also, prudent use of antibiotics and further research to alternative agents are warranted.

## Introduction

The increase of antimicrobial resistance is a major concern worldwide. Surveillance studies showed an increase in resistance for both Gram positive and Gram negative bacteria [1], including *Escherichia coli*, the most prevalent causative agent of urinary tract infections (UTIs) [1]–[4]. Moreover, the prevalence of extended spectrum beta-lactamase (ESBL) and/or carbapenemase producing multi drug resistant *E. coli* clones increased not only in the hospitals but also in the general population [1]–[4]. For the treatment of UTIs antibiotics are often prescribed empirically. An incorrect empiric choice is, in combination with the high prevalence of UTIs, a serious risk factor for the increase of antibiotic resistance among uropathogenic *E. coli* [5] and favors the spread of resistant clones such as *E. coli* ST131 [6], [7]. Differences in the level of antibiotic resistance and the prevalence of ESBL producing strains exist between health care institutions within and between countries [1], [8], [9]. Since micro-organisms do not recognize national borders, cross border spread is likely to occur. This is also the case in the Euregion Meuse-Rhine (EMR), the border region of the Netherlands, Belgium and Germany. This Euregion is a densely populated area with considerable cross-border movement and cooperation between health care institutes [10]. Differences in antibiotic resistance and antibiotic treatment protocols as wel as in infection control policies pose a serious risk for patient transfer between health care institutions within and between countries. Current data of prevalence of antibiotic resistance including the prevalence of ESBL producing *E. coli* strains will guide physicians in their choice of adequate empiric treatment. Since such ward specific resistance data for patients visiting the urology services are hardly available we conducted a surveillance to determine the prevalence of (multi drug) resistant *E. coli* isolates including ESBL producing strains collected from patients visiting a urology service in the Euregion Meuse-Rhine.

## Materials and Methods

### Ethics Statement

All bacterial isolates in this study were collected and analysed anonymously. Therefore, consent from the patient was not required and ethical approval was waivered. This is in agreement with the code for proper use of human tissue as formulated by the Dutch Federation of Medical Scientific Societies and the policy of the Medical Ethics Committee of het Maastricht University Medical Centre (MUMC).

### Bacterial Isolates

During a 6 month collection period between 2009 and 2011 unique, unrelated, non duplicate, consecutive isolates from urine samples from patients attending the urology services were collected.

A total of 421 *E. coli* isolates were included in this collection: 176, 126 and 119 from the Dutch, Belgian and German urology services, respectively. The following hospitals participated in the study: MUMC – Maastricht, Atrium Medical Centre – Heerlen and Viecuri Medical Centre – Venlo in the Netherlands, General Hospital Vesalius – Tongeren, Jessa Hospital – Hasselt and Centre Hospitalier Universitaire – Liège in Belgium, and Hermann-Josef Hospital – Erkelenz, St. Antonius Hospital – Eschweiler and St. Marien Hospital – Düren in Germany. Only one isolate per patient was included. Clinical data were not available. The isolates were collected and identified at the local laboratories according to standard microbiological methods, stored and sent to the MUMC for susceptibility testing.

### Antibiotic Susceptibility Testing

Quantitative susceptibility testing was performed using a microbroth dilution with Mueller-Hinton II cation-adjusted broth (Becton-Dickinson, Sparks, MD, USA) and micro titre plates with freeze-dried antibiotics (MCS Diagnostics BV, Swalmen, the Netherlands). The minimal inhibitory concentration (MIC) was defined as the lowest concentration showing no growth after 18 hours of incubation at 35°C. *E. coli* ATCC 35218 and ATCC 25922 were used as control strains. The MIC data were analysed using breakpoints defined by the European Committee on Antimicrobial Susceptibility Testing (EUCAST) [11]. Intermediate results were considered resistant. Multi drug resistance (MDR) was defined as resistance to three or more classes of antibiotics excluding the broad spectrum penicillins without a beta-lactamase inhibitor.

### Molecular Characterization of Beta-lactamases

Putative ESBL producing isolates (i.e. MIC for cefotaxime or ceftazidime > = 2 mg/L) were confirmed as an ESBL producer with a combination disk diffusion test [12] and characterized for the presence of TEM, SHV and/or CTX-M beta-lactamases with a micro-array (Check-points, Wageningen, the Netherlands). Based on these results *bla*_TEM_ or *bla*_CTX-M_ were amplified with PCR and specific primers [13], [14]. After purification (Spin PCRapace, Invitek, Berlin, Germany) automated sequencing was performed with the 3730 DNA analyzer with BigDye Terminator v1.1 (Applied Biosystems, Forster City, CA, USA).

### 2.4 Pulsed Field Gel Electrophoresis (PFGE) and Multi locus Sequence Tying (MLST)

All MDR isolates and ESBL producing isolates were further analyzed with PFGE [15]. All isolates with a indistinguishable or closely related pulsotype [16] and all ESBL producing isolates were analyzed with multi locus sequence typing (MLST) using the scheme specified at the University College of Cork *E. coli* MLST web site [17].

### 2.5 Empiric Treatment

The appropriateness of amoxicillin-clavulanic acid, piperacillin-tazobactam, cefuroxime, ceftazidime, cefixime, ceftibuten, ciprofloxacin, nitrofurantoin, trimethoprim-sulfamethoxazole and gentamicin for empiric treatment was determined, These agents are considered representatives to other antibiotics in their class.

As criterion for appropriateness of empiric treatment for complicated UTI, a 10% resistance cutoff value was used [18]. If the prevalence of resistance for a specific agent was higher than 10%, this agent was considered not appropriate choice for empiric treatment.

### Statistical Analysis

A Pearson’s chi square test or Fischer’s exact test was performed to determine statistically significant differences of resistance between the three different countries (subregions) of the Euregion Meuse-Rhine (SPSS-software, version 18.0, IBM, Armonk, NY, USA). A modified false discovery rate (FDR) method developed by Benjamini and Yekutieli was used as correction for multiple testing [19]. A p-value <0.05 was considered statistically significant.

## Results

Significant differences in prevalence of antibiotic resistance between the three regions in the Euregion were found for several antimicrobial agents tested including the fluoroquinolones, and folate antagonists (Table 1). Overall, resistance was highest among the Belgian isolates and lowest among the Dutch strains. Amoxicillin-clavulanic acid resistance ranged from 24% in the Netherlands to 39% in Belgium (p = 0.018). Resistance to piperacillin-tazobactam was still low (1–9%) with the highest prevalence of resistance among the Belgian isolates (p = 0.014). Among the Belgian isolates resistance to cefuroxime, cefotaxime, ceftazidime was higher compared with resistance among the Dutch isolates (p<0.004, p = 0.097 and p = 0.097, respectively). The prevalence of resistance to the fluoroquinolones ranged from 20% among the Dutch isolates to 40% among the Belgian ones (p< = 0.007). Significant difference in resistance between the Dutch isolates (20%) and Belgian isolates (40%, p = 0.018) was observed for the folate antagonists. Resistance to the carbapenems was not demonstrated.

**Table 1:** Antimicrobial resistance among (%) *E. coli* isolates.

Antibiotic agent	NL n = 176	B n = 126	G n = 119	NL vs B p-value	NL vs G p-value	B vs G p-value
Amoxicillin	48	60	49	–	–	–
Amoxicillin-clavulanic acid	24	39	27	0.018	–	–
Piperacillin	43	56	50	–	–	–
Piperacillin-tazobactam	3	9	1	–	–	0.014
Cefuroxime	5	17	10	<0.004	–	–
Cefotaxime	3	10	8	–	–	–
Ceftazidime	3	10	7	–	–	–
Cefixime	7	13	8	–	–	–
Ceftibuten	5	8	5	–	–	–
Cefepime	2	7	5	–	–	–
Ciprofloxacin	20	37	29	0.007	–	–
Norfloxacin	24	44	33	<0.004	–	–
Levofloxacin	19	37	29	<0.004	–	–
Moxifloxacin	20	39	32	<0.004	–	–
Nitrofurantoin	5	5	2	–	–	–
Trimethoprim	22	39	32	0.011	–	–
Trimethoprim-sulfamethoxazole	21	36	31	0.018	–	–
Amikacin	0	4	0	0.043	–	–
Gentamicin	5	6	12	–	–	–
Tobramycin	6	9	13	–	–	–

NL  =  the Netherlands, B  =  Belgium and G  =  Germany, -  =  not significant.

Putative ESBL producing isolates were found in 10, 14 and 9 isolates among the Dutch, Belgian and German isolates, respectively. ESBL production was confirmed for 20 isolates (i.e. 3, 8 and 9) resulting in a prevalence of ESBL producing isolates of 1.7%, 5.5% and 7.6% among the Dutch, Belgian and German isolates, respectively (p = 0.043 for the Dutch and German isolates). The confirmed ESBLs were mainly CTX-M 15 (Table 2).

**Table 2:** Number and identification of ESBL producing isolates.

	Netherlands	Belgium	Germany
CTX-M 1	0	2	1
CTX-M 14	1	1	0
CTX-M 15	2	1	8
CTX-M 55/79	0	2	0
TEM 52	0	1	0
**Total**	**3 (1.7%)**	**7 (5.5%)**	**9 (7.6%)** *

* Significantly higher than among isolates from the Netherlands (p<0.05).

**Figure 1:**
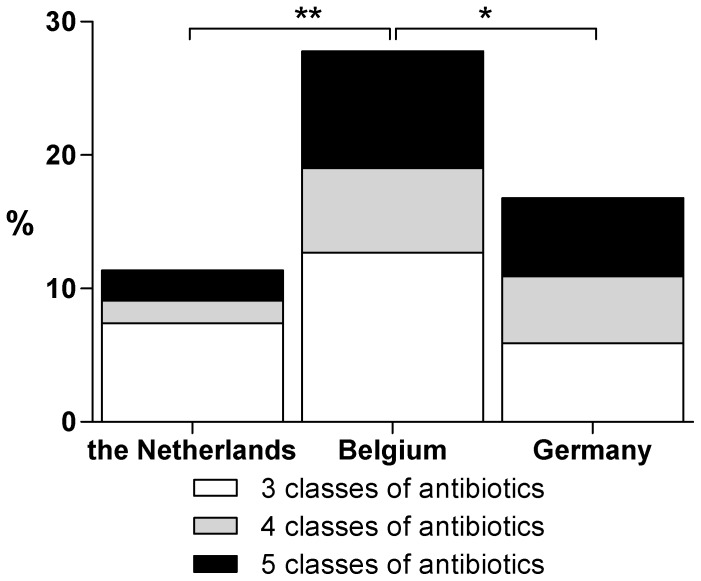
Prevalence of multi drug resistance of *E. Coli.* *  =  p<0.05, **  =  p< = 0.001.

MDR was observed in 74 out of 421 isolates. The prevalence of the MDR isolates ranged from 11% (n = 20) in the Dutch subregion to 27% (n = 34) in the Belgian subregion (p< = 0.001) (Figure 1).The PFGE pulsotypes of 24 isolates of these 74 MDR isolates were found more than ones. *E. coli* ST 131 strain was the most prevalent one (63%) of which 53% was an ESBL producer divided over 3 different pulsotypes (A,C and D, Table 3). This ST was demonstrated in all three subregions. The second most prevalent pulsotype (B) (13%) consisted of 2 STs, where ST1394 is a single nucleotide polymorphism of ST393. Among the ESBL producing isolates ST131 was the also the most prevalent ST (47%).

**Table 3:** Pulsotypes and STs of multi drug resistant and/or ESBL producing isolates.

Pulsotype	ST (CC)	n	n ESBL	ESBL Types
A	131	10	5	CTX-M 14, 15
B	393 (31)	2	0	
	1394	1	0	
C	131	3	1	CTX-M 15
D	131	2	2	CTX-M 15
E	624	2	0	
F	162 (469)	2	2	CTX-M 55/79
G	88 (23)	2	0	
ESBL singletons	10 (10)	1		CTX-M 1
	744 (10)	1		CTX-M 1
	131	1		CTX-M 15
	23 (23)	1		TEM 52
	88 (23)	1		CTX-M 1
	224	1		CTX-M 15
	964 (405)	1		CTX-M 15
	2509	1		CTX-M 14
	648	1		CTX-M 15

ESBL singletons: ESBL strains with a pulsotype of which no similar type has been demonstrated among the other typed isolates.

ST  =  sequence type, CC  =  clonal complex, n  =  amount.

Only pulsotype A was demonstrated in all three subregions. The other pulsotypes were only observed in one subregion.

**Figure 2:**
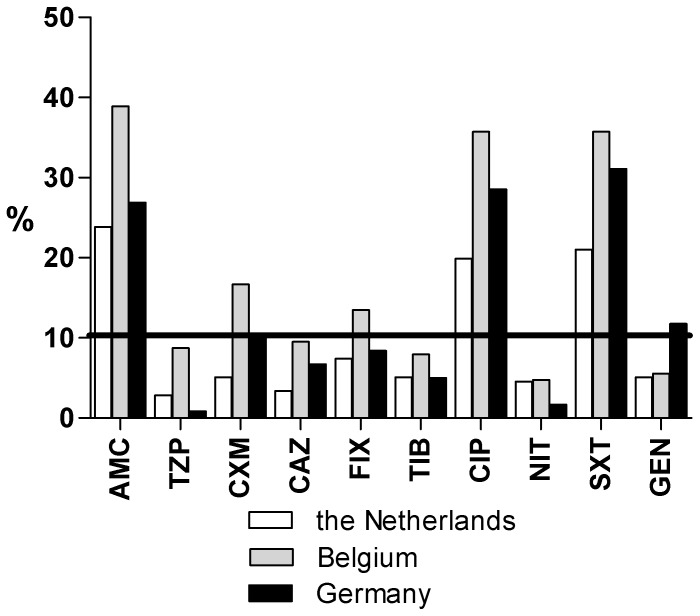
Antibiotic resistance and suitability of antibiotics for empiric treatment. Antibiotic resistance for the different antibiotics among the three groups of isolates compared with a 10% resistance cutoff to decide whether an antibiotic agent is suitable for empiric treatment. AMC  =  amoxicillin-clavulanic acid, TZP  =  piperacillin-tazobactam, CXM  =  cefuroxime, CAZ  =  ceftazidime, FIX  =  cefixime, TIB  =  ceftibuten, CIP  =  ciprofloxacin, NIT  =  nitrofurantoin, SXT  =  trimethoprim-sulfamethoxazole and GEN  =  gentamicin.

For empiric treatment of a complicated UTI, the prevalence of resistance should not exceed 10%. Taken into account this cutoff value several antibiotic agents are no longer appropriate for (oral) empiric treatment (Figure 2). These include amoxicillin-clavulanic acid, the fluoroquinolones and the folate antagonist in all three subregions and also some cephalosporines in the Belgian subregion. However, piperacillin-tazobactam and several third generation cephalosporines are still appropriate, which applies also for nitrofuratoin, although its pharmacokinetic properties do not support its use for a complicated UTI.

## Discussion

This study provides current data of the antimicrobial resistance of *E. coli* isolates collected from nine urology services in the Euregio Meuse-Rhine. We found significant differences in resistance between the Dutch, Belgian and German isolates. Although, overall the prevalence of resistance was highest among the Belgian isolates and lowest among the Dutch ones, the prevalence of ESBL producing isolates was highest among the German isolates.

More importantly, this study also indicates that most antibiotics used as first choice oral empiric treatment for UTIs (amoxicillin-clavulanic acid, fluoroquinolones, trimethoprim-sulfamethoxazole) [20] are not appropriate for this purpose since the prevalence of resistance exceeds 10% [18]. Previously, it has been demonstrated that the prevalence of antimicrobial resistance from invasive clinical isolates was higher in Belgium and Germany than in the Netherlands [1]. but has, to our knowledge, not been described for isolates from the urology services. This study also demonstrates that the globally emerging *E. coli* ST131 [6], [7], [21], [22] was prevalent in this entire population and also frequently produced an ESBL.

The *E. coli* isolates were collected from urine samples from patients of both gender and all ages visiting the urology services in the participating hospitals of this cross border multi centre surveillance study to prevent selection bias. Since all isolates were tested in one centre, the results were not influenced by differences in methodology. Unfortunately, additional patient and clinical data were not available. Since we cannot specify the underlying illness of the patients, this should be taken into consideration when initiating empiric treatment.

Comparing the resistance rates of *E. coli* from the EARSS annual report 2009, which consist mainly of isolates from invasive disease [1], and this study, the prevalence of resistance was comparable, except for resistance to the fluoroquinolones. This was higher among the urology isolates (11% vs. 19–20% for the Dutch isolates, 20% vs. 37–45% for the Belgian isolates and 23% vs. 29–33% for the German isolates, Table 1). These differences might be related to the high prescribing rate of fluoroquinolones at the urology services [8], [23].

Moreover, differences in antibiotic use [24] are also the most likely reason for the differences in resistance between the three countries in this study, although, differences in patient population can also play a role.

Compared with prevalence of resistance among general practice (GP) patients [2], [3], [9], [25] the resistance in this study was much higher. This is probably due to the more frequent antibiotic use among urology patients compared with GP patients, the higher prevalence of urinary tract comorbidities and the use of catheters, which might select for resistant strains [5], [26], [27]. Compared to a previous study among urology isolates the Dutch isolates in the present study showed a lower prevalence of resistance for the folic acid antagonists and higher resistance for the quinolones [8]. Taken into account that only antimicrobial agents with a resistance level of 10% or less are suitable for empiric therapy [18], fluoroquinolones, broad spectrum penicillins and folate antagonist are not an appropriate choice. This applies for the whole Euregion. Alternatives agents are limited and include nitrofurantoin, which is not used for complicated UTIs because of poor tissue penetration [28], piperacillin-tazobactam, which cannot be administered orally, and the third generation cephalosporines. However, high use of cephalosporines could increase the selection and persistence of ESBL producers and should be kept to a minimum [29]. Therefore, further research to potential alternatives, such as fosfomycin, is essential.

During the last years (multi drug) resistant strains have become a major healthissue. National and international surveys show an increase in nosocomial and community acquired infections involving ESBL and/or carbapenemase producing isolates [1], [2], [30], [31]. Also, globally the MDR and often ESBL producing *E. coli* ST131 clone is emerging [6], [7], [21], [22]. An infection with these isolates can often not be treated with penicillins, cephalosporines, fluoroquinoles and folate antagonists [20], [32]. Consequently, adequate antibiotic therapy is delayed because of inadequate empirical therapy, which affects the patient outcome negatively [33]. In this study the percentage of ESBL producing isolates was less than 8% in all three subregions, with the lowest prevalence of ESBL producers among the Dutch isolates. Most ESBL producers contained the ESBL type CTX-M, which is prevalent [21], [22], [29]. The high prevalence of ST131 among the tested isolates is a point of concern, since this clone can easily acquire more resistance traits [6], [7], [21] and will become a major health risk since it is infesting itself in our population. However, current prevalences of (multi drug) resistance do not justify alteration of the treatment protocols to empiric therapy with carbapenems. Nevertheless, continuous ward specific surveillance is necessary to monitor changes in antibiotic resistance MDR strains. Moreover, further research is indicated to find alternative antibiotic agents of which fosfomycin is a possibility. This agent has high tissue penetration, maintains active against ESBL producing isolates [34], but is until now not registered for complicated UTI. Therefore, further research into the usefullness of fosfomycin for treatment of this type of infection is warranted.

Concluding, ward specific surveillance of antimicrobial resistance is important to monitor resistance over time on a regional, national and international level since we demonstrated significant differences in antibiotic susceptibility between the three subregions in the Euregion Meuse-Rhine. We also found a high prevalence of *E.coli* ST131 and CTX-M type ESBLs, suggesting a spread of this clone in the entire Euregion.

Due to the high prevalence of resistance many antibiotics including amoxicillin-clavulanic acid, the fluoroquinolones and the folate antagonists are no longer suitable for empiric treatment of complicated UTI, therefore, further research to other possible agents is needed.
